# Editorial: Biosynthesis, purification, characterization and uses of natural compounds in plants

**DOI:** 10.3389/fpls.2023.1162676

**Published:** 2023-07-06

**Authors:** Monica Butnariu, Zipora Tietel

**Affiliations:** ^1^ Chemistry & Biochemistry Discipline, Life Sciences University “King Michael I” from Timisoara, Timis, Romania; ^2^ Food Science, Agricultural Research Organization, Rishon LeZion, Israel

**Keywords:** natural products, biochemical pathways, secondary metabolites, chemical analysis, health properties

Natural products impart immense chemical diversity to the plant kingdom, owing to more than 200,000 specialized metabolites (SM), many of which are derived from phenylpropanoid, terpenoid and alkaloid biosynthetic pathways (Sousa Silva et al., 2019). Furthermore, these molecules are distinguished on the basis of their modifications such as glycosylation, acylation, and prenylation (Wu and Lei, 2022). Aromatic and medicinal plants serve as abundant sources of natural products, many of which possess significant benefits for human health. These benefits include as the prevention and/or treatment of various cancers, as well as inflammatory, cardiovascular and neurodegenerative diseases (Che and Zhang, 2019). Additionally, these SM exhibit antimicrobial, antiviral and antidiabetic properties (Mohammed and Khan, 2022).

Apart from species and/or organ specific accumulation patterns, natural products occur in response to various abiotic and biotic stresses. They serve diverse biological functions, acting as attractants, repellants and other olfactory signaling molecules involved in olfactory communication. Additionally, they play an essential role in growth and development (Weng et al., 2021). In certain cases, these natural products are localized within specific tissues and/or specialized cells such as leaf epidermal trichomes (Liu et al., 2019). To harness the potential of plant natural products for human health and the agri-food industries it is crucial to gain in-depth understanding of their biosynthetic pathways and bioactivities. However, due to limited knowledge regarding the genetic factors influencing natural product production in plants, strategies for feasible extraction and purification from plants are required. This task presents a considerable challenge due to typically low abundance of SM in plants.

*Dendrobium officinale*, a traditional medicinal herb and a new functional food (Chen et al., 2021), still holds many mysteries regarding its phytochemical composition, particularly its bioactive alkaloids The limited knowledge can be attributed, in part, to the absence of robust analytical techniques. In their work, Song et al. developed an advanced SPE-LC-MS/MS chromatographic method for *D. officinale* analysis, used to thoroughly profiling plant extracts. A special emphasis was given to alkaloids, describing their chemical structures and main characteristic fragments. In addition, they showed that methyl jasmonate elicits SM biosynthesis in *D. officinale* through defense-related gene expression. Plant’s profile and particular methods will facilitate its utilization for nutritional and medicinal purposes.

Essential oils (EO) are complex mixtures of volatile SM, often possessing powerful bioactivities, such as antioxidant properties, hepatoprotective effects, and the ability to promote wound healing (Mohammed et al., 2021; Mohammed et al., 2022). Recently, an α-amylase inhibition and antibacterial bioactivities were reported for *Sabina chinensis* leaf EO (Gu et al., 2018). The work by Zhang et al. optimized the extraction process, assessing *S. chinensis* largely unknown chemical composition by GC-MS and showing mainly terpenes as the main constituents, with in-year composition variability. In addition, they established its reported antifungal activity against two *Fusarium* species. By employing OPLS-DA, the authors identified specific compounds ascribed for the antifungal bioactivity, thereby expanding our knowledge of its potential as a broad-spectrum antifungal agent with widespread applications.

Cuticular waxes, which consist of a mixture of alkanes and fatty esters, constitute leaf’s primary protective barrier against environmental stresses, playing an essential role in stress response and consequently influenced by it (Lewandowska et al., 2020). Speckert et al. addressed the open question of trees’ wax biosynthesis regulation in response to environmental conditions. They investigated the ongoing formation of wax constituents by conducting a ^13^CO2 pulse-chase labelling of sun-exposed vs. shaded branches of a mature beech tree during the late summer. Showing an ongoing fatty acid and SM *de-novo* biosynthesis, their results strongly suggest that trees adjust their lipid production and composition for acclimating to micro-environmental changes. These findings imply trees’ ability to adjust their wax production to varying environmental conditions, also providing valuable hints regarding resource allocation.

Prenylated chalcones have recently gained recognition as potent food components and dietary supplements, offering substantial health benefits, e.g., in cancer prevention and treatment (Venturelli et al., 2016). Membrane-bound prenyltransferases (PT) are involved in their biosynthesis from chalcones, which encouraged Guo et al. to attempted to construct the heterologous biosynthetic pathway of isobavachalcone in tobacco (*Nicotiana tabacum*). Isobavachalcone biosynthesis was successfully achieved using tobacco transient expression with exogenous isoliquiritigenin as a substrate, with *Humulus lupulus* (HL) and *Sophora flavescens* (SF) PT homologs. Furthermore, *de novo* biosynthesis of isobavachalcone in transgenic tobacco lines was accomplished *via Glycine max* genes generating endogenous isoliquiritigenin, with SF PT on the multigene vector. This work emphasizes the current need of leveraging plant metabolism to elegantly produce potent pharmaceuticals.

Rice (*Oryza sativa*) is the world’s most important food crop, and hence its grain yield of major significance. The work by Um et al. sheds light over the reciprocal relationship between actin-related proteins and gibberellic acid (GA) signaling and biosynthesis, both being key factors affecting growth and productivity. Plant height and growth were diminished by an *O. sativa* GA dwarf gene knockout, due to low levels its actin-related protein, as well as GA. GA application restored plant height. Overexpression of that gene also resulted in normal phenotype. This work’s novelty lies in determining the central role of *O. sativa* GA dwarf gene in cell development and expression of elongation-related genes, also revealing the inter-relations with GA homeostasis, hence regulating plant development.

## Author contributions

MB—conceptualization, first draft writing, and advanced draft editing. ZT—conceptualization and advanced draft editing. All authors contributed to the article and approved the submitted version.
